# Enhancing Healing in Hidradenitis Suppurativa With Tobacco Pouch Suture After CO_2_ Laser Excision Treatment

**DOI:** 10.1111/ijd.17679

**Published:** 2025-02-07

**Authors:** Martina Mussi, Michelangelo La Placa, Valeria Gaspari, Antonio Russo, Davide Melandri, Bianca Maria Piraccini, Corrado Zengarini

**Affiliations:** ^1^ Dermatology Unit IRCCS Azienda Ospedaliero‐Universitaria di Bologna Bologna Italy; ^2^ Department of Medical and Surgical Sciences Alma Mater Studiorum University of Bologna Bologna Italy; ^3^ SOC Centro Grandi Ustionati/Dermatologia Cesena (Forlì) AUSL Romagna Cesena Italy; ^4^ Dermatology Unit AUSL Romagna Forlì Italy

**Keywords:** hidradenitis suppurativa (HS), laser, tobacco pouch suture, treatment

**Abbreviations**
CO_2_
: carbon dioxide
HS: hidradenitis suppurativa

While medical therapies are the mainstay for early stages, treating advanced hidradenitis suppurativa (HS) poses challenges due to the frequent recurrence of lesions, which often requires wider surgical excisions [1]. This can lead to delayed wound healing, an increased risk of complications and scarring, and significant patient discomfort, creating a notable barrier to surgical management [2]. Carbon dioxide (CO_2_) laser treatment has proven to be an effective modality for reducing recurrence and improving outcomes compared to traditional surgery [3]. However, further refinements in this technique are still needed to enhance its overall effectiveness, as well as wound management after surgical intervention [4].

We propose to use a tobacco pouch suture following CO_2_ laser debulking to enhance wound margin approximation and accelerate healing. This technique involves placing a continuous purse‐string suture around the wound's circumference, drawing the edges inward, reducing the wound area, and minimizing tension, which lowers the risk of dehiscence (Figure 1). In our experience, this method led to faster granulation and full re‐epithelialization compared to other treatments that did not incorporate this specific suture technique, resulting in minimal scarring and significantly reducing patient discomfort and pain during recovery.

**FIGURE 1:**
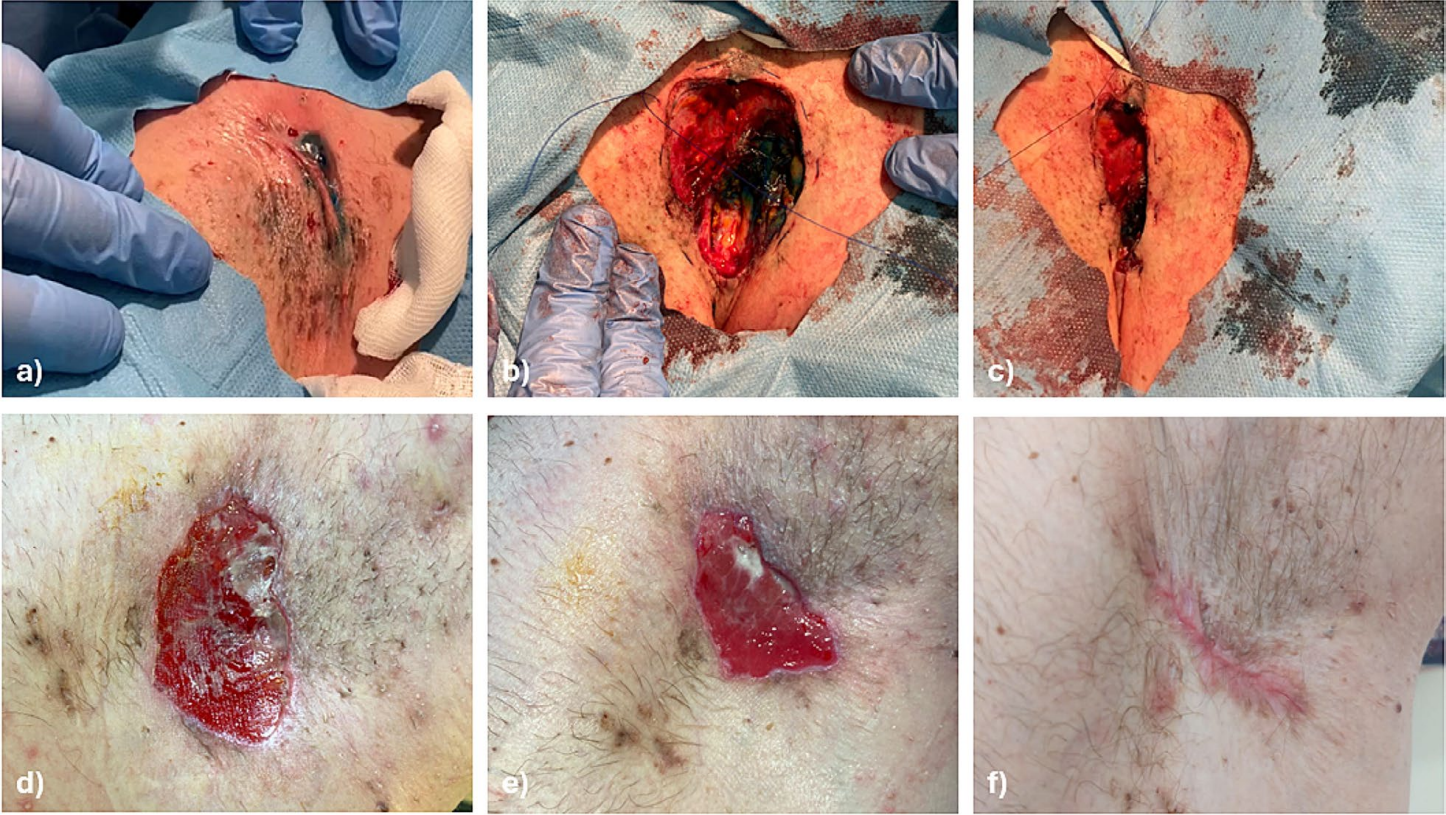
(a) Preoperative view of the left axillary cavity showing active inflammation and fistulas colored with Blue Patent staining to identify the course and the depth of the sinus tracts; (b) intraoperative view following CO_2_ laser excision of diseased tissue with application of the tobacco pouch suture around the margins; (c) closure of tobacco pouch suture technique which allows bringing wound margins closer, when suture knot applied; (d) first postoperative result after one week, showing excellent and rapid granulation of the tissue, with no signs of infection or wound dehiscence which allowed to remove the suture; (e) two‐week follow‐up, showing that the collagen‐based dressing had been almost fully reabsorbed and that the size of the wound has reduced; (f) three‐month follow‐up, with full re‐epithelialization and complete healing of the wound, leaving minimal scarring.

As a representative case, we present a 35‐year‐old male suffering from HS since the age of 12, who progressed to Hurley Stage III, International Hidradenitis Suppurativa Severity (IHS4)‐score 13 [5], with HS of the left axilla with refractory lesions unresponsive to antibiotics, biologics, and topical therapies. Treatment with adalimumab was discontinued after 13 months of administration for the development of anti‐TNF‐alpha antibodies. Given the lack of response to medical therapies, ablative surgical management was pursued, and CO_2_ laser excision was performed under local anesthesia after 2 months of adalimumab suspension. Guerbet blue patent V staining was previously injected into the sinus tracts to delineate their course and depth accurately, guiding the excision of diseased tissue (Figure 1a,b). Both continuous and SmartPulse modes of the DEKA CO_2_ laser were utilized, with power settings ranging from 2 to 5 watts for the continuous mode and 30 to 45 W for the SmartPulse mode. Following laser debulking, the wide surgical defect created significant wound tension.

To address this, we employed a tobacco pouch suture technique, a continuous purse‐string suture around the wound's circumference, with 3‐0 prolene, which reduced tension and promoted wound edge approximation (Figure 1b,c). A collagen‐based dressing was applied, followed by sterile gauze and bandage. Postoperatively, the patient was medicated every 48 hours with betadine‐based antiseptic gauzes and bandages and received only on‐demand oral administration of anti‐inflammatory drugs. He exhibited rapid tissue granulation, with the suture removed after 1 week due to excellent progress (Figure 1d). At 2 weeks, the wound demonstrated full re‐epithelialization, and at 3 months, complete healing with minimal scarring was observed (Figure 1e,f). The patient reported significant symptom relief and a marked improvement in quality of life, expressing great satisfaction with the outcome. CO_2_ laser excision is an established method for HS management, particularly in cases where traditional excision risks delayed healing due to the large defect size.

The addition of the tobacco pouch suture technique offers advantages such as reduced wound tension and faster healing, as in this case. This combined approach demonstrates the potential for better surgical outcomes in HS management, especially in areas prone to significant functional and cosmetic impact. In conclusion, the integration of CO_2_ laser excision with the tobacco pouch suture technique proved highly effective in treating severe axillary HS. This method facilitated wound closure, reduced healing time, and minimized scarring, underscoring its value as a surgical option for complex HS cases. Further studies are warranted to validate this approach and establish standardized protocols for its application.

## Consent

The patients in this manuscript have given written informed consent to the publication of their case details.

## Conflicts of Interest

The authors declare no conflicts of interest.
